# Analysis of the evolution trends and influential factors of bankfull discharge in the Lower Yellow River

**DOI:** 10.1038/s41598-022-24310-6

**Published:** 2022-11-21

**Authors:** Meng Chen, Linjuan Xu, Chunpeng Xing, Haifan Xu, Wanjie Zhao

**Affiliations:** 1 North China University of Water Resources and Electric Power, School of Water Conservancy, Zhengzhou, 450046 China; 2 Key Laboratory of Lower Yellow River Channel and Estuary Regulation, MWR, Yellow River Institute of Hydraulic Research, YRCC, Zhengzhou, 450003 China; 3 College of Water Conservancy and Hydropower Engineering, HoHai University, Nanjing, 210098 China

**Keywords:** Hydrology, Natural hazards

## Abstract

After the Xiaolangdi Reservoir (XLDR) was put into operation, new water and sediment conditions have improved the silting condition of the Lower Yellow River (LYR), but against this background, the flow capacity of the LYR has become more complex. In this research, the measured water and sediment data of seven hydrological stations in the LYR from 1950 to 2020 were systematically collated. The evolution trend and development period of the bankfull discharge in this area were studied based on the wavelet analysis method, and the main factors influencing the evolution of the bankfull discharge were explored. The results indicate that the evolution process of bankfull discharge in the LYR has experienced two phases in the last 70 years. XLDR has been impounded since October 1999. Before XLDR operation, the bankfull discharge of the LYR has a main time scale of about 26 years. After XLDR operation began, it has a time scale of about 10 years. The bankfull discharge of the LYR shows two phases of evolution, and these phases are mainly influenced by the factors of water and sediment conditions. This research is needed for a deeper understanding of flow-bed and river discharge and sediment transport capacity under the water and sediment conditions in the LYR.

## Introduction

Bankfull discharge refers to the flow at ordinary times between the water level and the flood plain. The corresponding flow rate is high, sediment transport capacity is high, and bed-making function is strong. Bankfull discharge is an important index reflecting the capacity of the water-making bed and the flood discharge and sediment transport of the riverbed, and it is also an important research subject in riverbed evolution^1^. It is an important indicator reflecting the bed forming capacity of water flow and the flood and sediment discharge capacity of a river channel. Since operation of the Xiaolangdi Reservoir (XLDR) began, the bankfull discharge and sediment transport capacity of the Lower Yellow River (LYR) has been restored. However, the annual scouring efficiency from 2015 to 2017 is significantly lower than that at the initial phase of XLDR operation^2^. After 2018, the phenomenon of silting and scouring of the main stream in the XLDR area gradually become apparent, and the change in the sediment carrying capacity of the flow is complex. Under this background, it is of great significance to research the changes in bankfull discharge and its influencing factors on the LYR.

The amount of water and sediment from the Yellow River has continuously changed in the past century^3^. The annual precipitation time series of Qinghai Province in the upper reaches of the Yellow River has periodic variation regularity in recent 63 years^4^. Since the mid-1970s, the significant periods of runoff and sediment discharge have changed discontinuously in the Middle Yellow River^5^, which has been weakening since 1980, especially for the sediment discharge, which basically disappeared after 2000^6^. The LYR is greatly affected by XLDR. Before the operation of the XLDR, the main channel is shrinking and the width and depth gradually decreased. Annual runoff and sediment concentration of the river from 1985 to 1996 show a significant downward trend with sudden change^7^. After the operation of the XLDR, the main channel is been scoured, and the width and depth increased^8^. At the same time, the water and sediment amount of the Yellow River Basin have shown significant downward trend^9^, which keeps the LYR in a continuous evolution and development process, and which is restricted by three elements, incoming water and sediment, river bed boundary, and estuary datum level^10^. For other river basins, after the operation of the Three Gorges Project, the Middle Yangtze River have been continuously scoured, bank collapse often occurs in local reach, and river regime is gradually unstable^11^. From the 1950s to the 2000s, the flow and sediment discharge of the Pearl River basin are also affected by human activities and climate change ^12^. The Wanggang mudflat, Jiangsu, China, between the abandoned Yellow River and the Yangtze River estuary, and provides the basis for a mechanistic understanding of the morphological evolution and development of predictive sediment transport and erodibility models ^13^.

According to the characteristic of water and sediment in the Yellow River, it is found that the bankfull discharge is closely related to water and sediment conditions ^14–18^, which is influenced by peak discharge, median particle size ^19^, riverbed boundary conditions ^20^, and the shape of mainchannel^21,22^. According to the influencing factors of bankfull discharge, two calculation methods of bankfull discharge have been proposed, such as the numerical simulation method and the comprehensive method. The numerical simulation method of a one-dimensional hydrodynamic model can determine the bankfull discharge in the LYR, and which has the characteristics of high prediction accuracy, but great difficulty in calculation^23^. The comprehensive method is that combined the geometric average of logarithmic transformation and the weighted average based on the distance between two adjacent sections^24–27^. This method takes into account more comprehensive factors, but the calculation process is complex.

In recent years, the amount of water and sediment of the LYR has continued to decline under the influence of many factors. Moreover, it has been found that the bankfull discharge is affected by many factors. However, from the results obtained, there are few studies based on the characteristics of the periodic change of bankfull discharge in the LYR under long-time series and determining the main influencing factors. In view of this, this research systematically collates the measured water and sediment data on the Huayuankou (HYK), Jiahetan (JHT), Gaocun (GC), Sunkou (SK), Aishan (AS), Luokou (LK), and Lijin (LJ) hydrometric stations of the LYR from 1950 to 2020. Based on the wavelet analysis method, this research analyzes the bankfull discharge variation characteristics of the above seven stations, explores the evolution trend and period of bankfull discharge, and deeply studies the main factors affecting the bankfull discharge evolution.

## Materials and methods

### Research area

**Figure 1:**
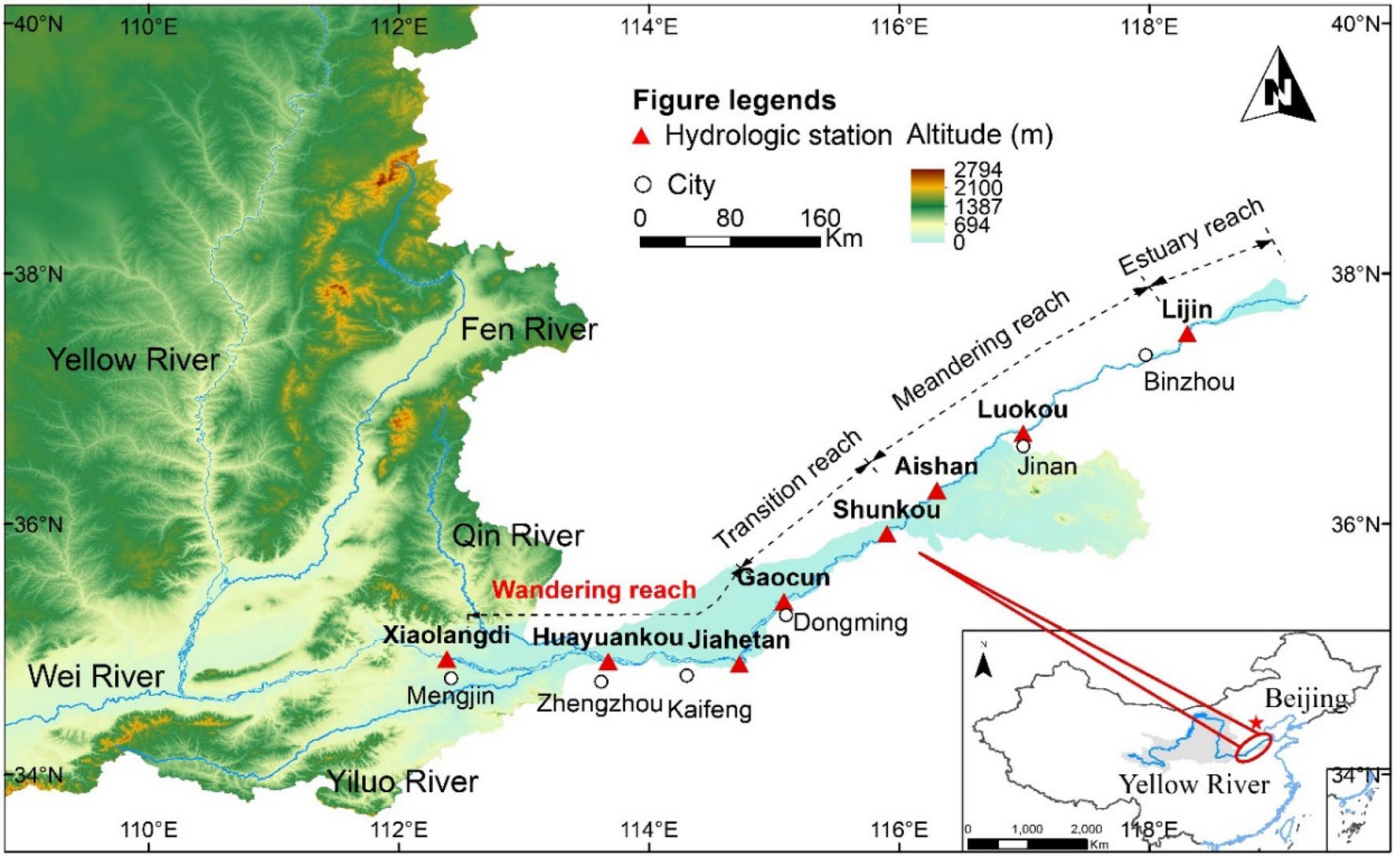
Map of the study area. Created by ArcGIS 10.2 software(https://www.arcgis.com/).

The LYR has a total length of 786 km, characterized by a wide and shallow upper section, a narrow and deep lower section, and great changes in scouring and silting. The longitudinal gradient of the river is between 0.172 and 0.265‰^28^. According to the morphological characteristics of the river course, the downstream river course is divided into a wandering section from Mengjin to Gaocun, a transition section from Gaocun to Taochengpu, a curved section from Taochengpu to Lijin, and an estuary section below Lijin. This research area is the LYR. There are seven hydrological stations along the reach, HYK, JHT, GC, SK, AS, LK and LJ. The plane distribution is shown in Fig. 1. The disharmonious distribution of water and sediment of the LYR has led to serious problems such as sediment deposition and flood control, which is the focus of the Yellow River.

### Research method

#### Data source

This research studies the evolution trend of bankfull discharge in the Lower Yellow River from 1950 to 2020. The data of discharge, sediment concentration, annual runoff, and annual sediment discharge are all from the China River Sediment Bulletin on the official website of the Ministry of Water Resources of the People's Republic of China. However, the official website of China Sediment Bulletin only announced that the lower reaches of the Yellow River will only be discharged with full reservoirs from 2017 to 2020. By collecting the measured water and sediment data of the LYR from 1950 to 2020, and using the new method for calculating the bankfull discharge proposed in^29^, the bankfull discharge of seven stations in the LYR from 1950 to 2020 was calculated. This method was used to calculate the bankfull discharge of the seven hydrological stations in the LYR from 2017 to 2020. This was compared with what was published in the Sediment Bulletin of rivers in China. The results show that the two sets of data are completely identical, which indicates that the method in^29^ is reliable for calculating bankfull discharge.

#### Data treatment method

Wavelet analysis can be used to carry out multi-scale detailed analysis on a signal and has a strict mutation point diagnosis ability in the mathematical sense^30^. When applying the theory of wavelet analysis to solve practical problems, it is the basis of wave-let analysis to select the appropriate basis wavelet function. Morlet wavelet can be used to analyze the time–frequency multi-resolution function^31^, which makes it possible to better study the problem of time series, clearly reveals a variety of change cycles hidden in the time series^32^, and fully reflects the change trend of the system in different time scales. In this research, Morlet wavelet was used as the basic wavelet function, and the time scale characteristics of bankfull discharge in the LYR were analyzed based on the Morlet continuous complex wavelet transform. The complex wavelet transform can provide both the phase and amplitude information of the time series, which is conducive to the further analysis of the bankfull discharge evolution process.

The basic principle of wavelet analysis is to obtain low-frequency or high-frequency information of the signal by increasing or decreasing the scale, analyze general or detailed information about the signal, and analyze different time scales and spatial local characteristics of the signal. The basic idea of wavelet analysis is to use a cluster of wavelet functions to represent or approximate a signal or function. Therefore, a wavelet function is the key to wavelet analysis. A wavelet is a wave with oscillation and rapid attenuation, which meets the condition that the integral is zero mathematically. The expression of wavelet function $\psi (t) \in L^{2} (R)$ is

1 $$\int_{ - \infty }^{ + \infty } {\psi (t)dt = 0}$$

where $\psi (t)$ is the base wavelet function or the mother wavelet function, and $L^{2} (R)$ is the spatial value with limited energy.

The wavelet sequence can be obtained by scaling the $\psi (t)$ base wavelet and translating the time axis to form a cluster function system $\psi_{a,b} (t)$. $\psi_{a,b} (t)$ is also called a sub-wavelet, and its expression is

2 $$\psi_{\alpha ,b} (t) = \left| a \right|^{ - 1/2} \psi \left( {\frac{t - b}{a}} \right)$$

where $a,b \in R,a \ne 0$, a is the scaling factor or scaling factor, reflecting the period length of wavelet, and b is the translation factor, reflecting the translation in time.

If $\psi_{a,b} (t)$ is a sub-wavelet given by Eq. (2), for a given energy limited signal $f(t) \in L^{2} (R)$, its continuous wavelet transform expression is

3 $$W_{f} (a,b) = \left| a \right|^{ - 1/2} \int_{R} {f(t)\overline{\psi }\left( {\frac{t - b}{a}} \right)} dt$$

where $W_{f} {(}a,b{)}$ is the wavelet transform coefficient, $f(t)$ is a signal or square integrable function, a is the expansion scale, b is the translation parameter, and $\overline{\psi } {(}\frac{x - b}{a}{)}$ is a complex conjugate function of $\psi (\frac{x - b}{a})$. The contour map of the real part of wavelet coefficients can show the distribution and phase change of signals with different time scales at different time (t) points.

After the time series are adjusted, the square values of all wavelet coefficients of different scales a are integrated in the b domain to obtain the wavelet variance $Var(a)$, which is expressed as

4 $$Var(a) = \int_{ - \infty }^{\infty } {\left| {W_{f} (a,b)} \right|}^{2} db$$

The wavelet variance diagram shows the variation process of wavelet variance with scale a, which can be used to determine the relative intensity of different scale disturbances in the signal and the main time scale, namely, the main period. The larger the extreme value, the greater the contribution to the wavelet variance, and the stronger the oscillation on the time series scale. The main period trend chart is the first main period wavelet coefficient change process of bankfull discharge evolution in the research area. It is drawn according to the results of the wavelet variance test and can reflect the average period and quasi oscillation change characteristics under the time scale of the main period.

## Results

### Variation trend of bankfull discharge in the LYR from 1950 to 2020

**Figure 2:**
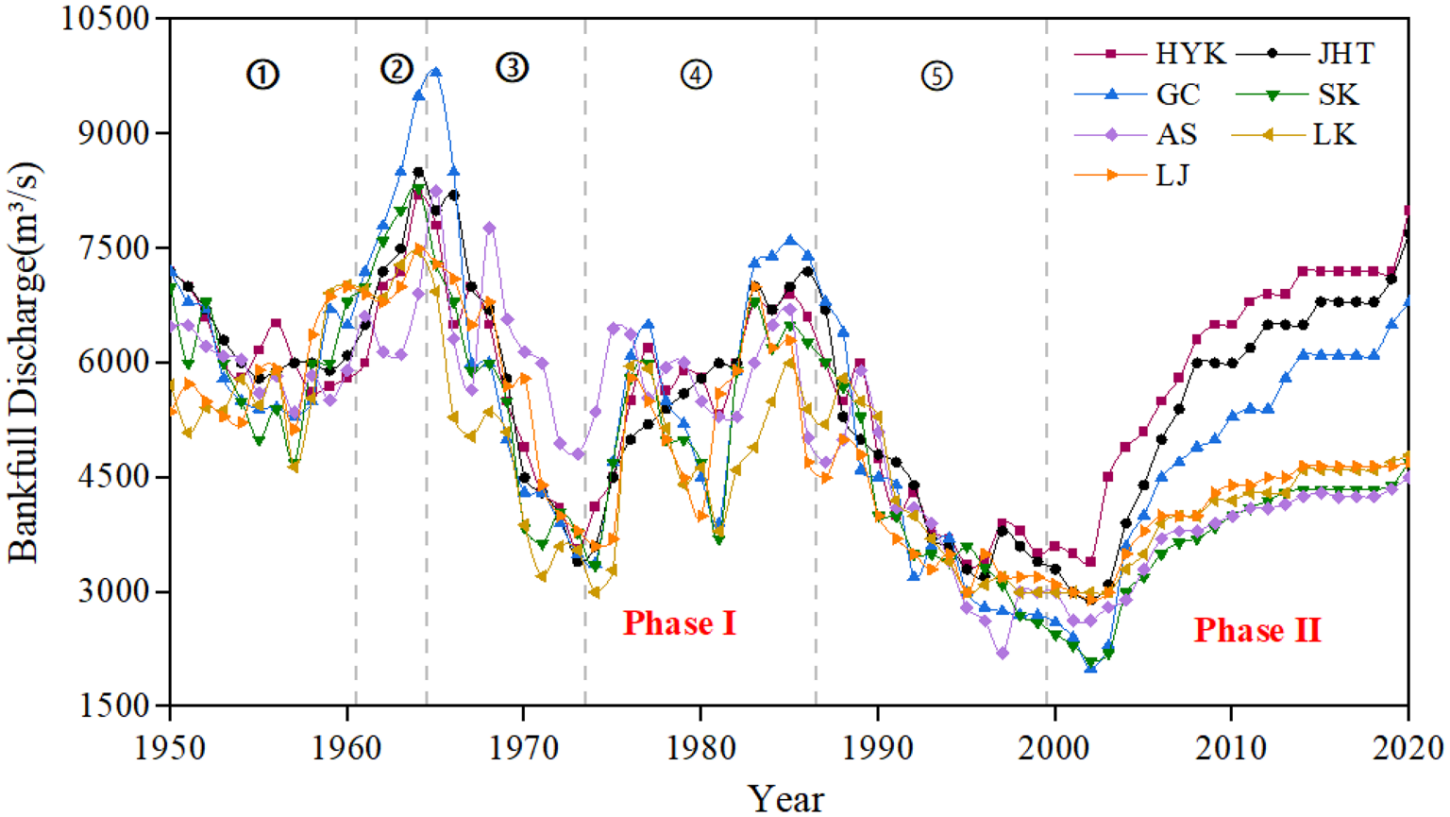
Variation process of bankfull discharge of the LYR from 1950 to 2020: ① 1950–1960, ② 1961–1964, ③ 1965–1973, ④ 1974–1986, ⑤ 1987–1999.

Figure 2 shows the change process of bankfull discharge of hydrological stations in the LYR from 1950 to 2020. It can be seen that, before the operation of the XLDR (1950–1999), the bankfull discharge evolution characteristics of the hydrological stations in the LYR showed an alternating increasing and decreasing trend in different ranges. After the operation of the XLDR began (2000–2020), it shows a continuous increasing trend. Thus, it is divided into two phases.

### Phase I: before XLDR operation (1950–1999)

According to the evolution characteristics of bankfull discharge in this phase, the phase can be divided into the following five periods. ① 1950–1960. The overall change in the bankfull discharge of the LYR was relatively stable, and the fluctuation range was relatively small. ② 1961–1964. The bankfull discharge of the LYR increased significantly year by year, and the bankfull discharge increased to the maximum value. ③ 1965–1973. The bankfull discharge of the LYR decreased as a whole and fluctuated slightly in each year. ④ 1974–1986. The bankfull discharge of the LYR showed an obvious alternating increasing and decreasing trend, with a large fluctuation range and a small overall increase. ⑤ 1987–1999. The bankfull discharge of the LYR showed an obvious linear de-creasing trend, and the bankfull discharge increased to the minimum value.

### Phase II: after XLDR operation began (2000–2020)

In this phase, the bankfull discharge of the LYR showed a slight decrease trend in the first two years, and the bankfull discharge of these seven hydrological stations then continued to increase. The details are shown in Table 1.

**Table 1:** Average bankfull discharge of each phase in the LYR.

Phase	Bankfull Discharge(m^3^/s)	Hydrological Station						
		HYK	JHT	GC	SK	AS	LK	LJ
Phase I	Max	8200	8500	9800	8300	8200	7460	7500
	Min	3560	3600	3370	3350	3560	3000	3600
	Average	5580	5620	5550	5270	5470	4900	5200
Phase II	Max	8000	7700	6800	4700	4500	4800	4700
	Min	3400	2900	2000	2100	3400	3000	2900
	Average	6070	5560	4840	3690	3760	4020	4010

Table 1 shows the bankfull discharge of each value in two stages before and after operation of the XLDR began in the Lower Yellow River from 1950 to 2020. It can be seen that, except for the increase in bankfull discharge in the second phase of HYK, the average of the other six hydrometric stations in the second stage is lower than that in the first stage.

### Evolution period of bankfull discharge based on wavelet analysis

The construction of the XLDR has artificially changed the inflow and sediment conditions of the LYR. The new water and sediment conditions have exerted a new influence on the scouring and silting evolution of the LYR, and the bankfull discharge has changed accordingly. Wavelet analysis was used to study the evolution of the bankfull discharge of the LYR before (1950–1999) and after (2000–2020) XLDR operation began.

### Evolution characteristics of bankfull discharge prior to XLDR operation (1950–1999)

**Figure 3:**
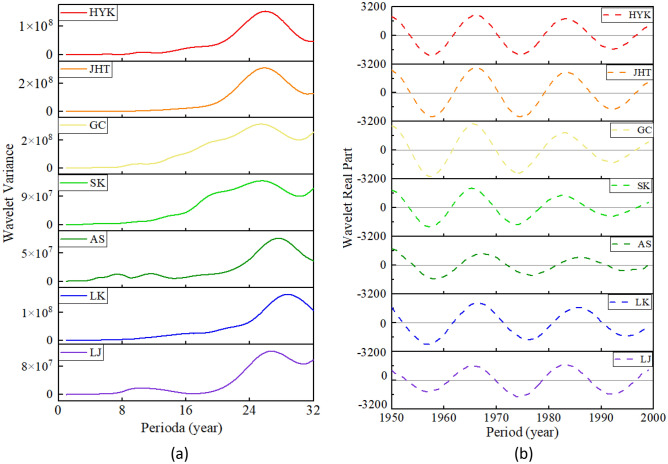
Variance diagram of wavelet coefficients for bankfull discharge of the LYR from 1950 to 1999: (**a**) wavelet variance, (**b**) main period trend.

Figure 3 shows a variance diagram of the wavelet coefficients of the bankfull discharge at these seven hydrological stations of the LYR from 1950 to 1999. Figure 3a is a variance diagram of the bankfull discharge in the LYR from 1950 to 1999, which reflects the main period of bankfull discharge evolution in the LYR during this period, and the corresponding main period is a time scale. Figure 3b is a trend diagram of the main period of the bankfull discharge, which reflects the change in the real part of the wavelet coefficients of the bankfull discharge during the first main period, and defines the period under the first main period from 1950 to 1999 in the LYR.

In Fig. 3a, it can be seen that there is one obvious peak in the wavelet variance map of the bankfull discharge of the LYR from 1950 to 1999. The maximum peak, also called the first main period, corresponds to a time scale of about 26 years. These seven hydrological stations have an unnoticeable second peak in different time scales, which is the second main period. It can be seen in Fig. 3b that, under the time scale of the first main period, the evolution period of the bankfull discharge in the LYR is about 18 years, and it has experienced quasi-three oscillations. The evolution period of the bankfull discharge from 1950 to 1999 of the LYR is shown in Table 2.

**Table 2:** Evolution period of bankfull discharge in the LYR from 1950 to 1999.

Period (years)	Hydrological Station						
	HYK	JHT	GC	SK	AS	LK	LJ
First primary period	26	26	25	25	28	29	26
Period under the first main period	18	18	18	17	18	19	18

**Figure 4:**
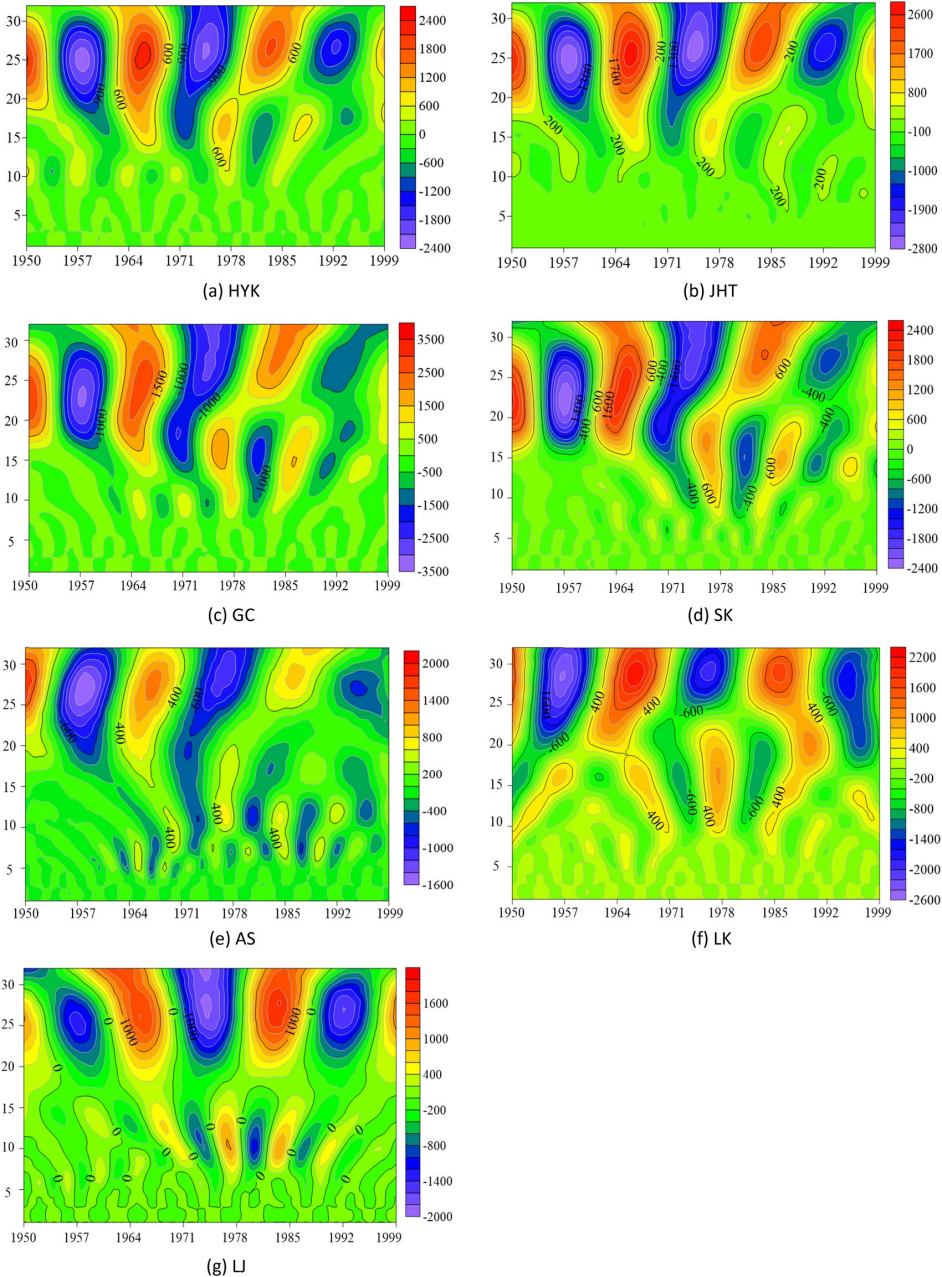
Real contour map of wavelet coefficients for the bankfull discharge of the LYR from 1950 to 1999. Created by MATLAB software (https://www.mathworks.com/products/matlab.html) and SUFER15 software (https://www.goldensoftware.com/products/surfer).

The real isoline map of the wavelet coefficient reflects the distribution of the bankfull discharge sequence in time scale and the periodic variation in different time domains. It also predicts the evolution trend of bankfull discharge in different time scales. In order to more accurately reflect the role of the real-part isoline map of wavelet coefficients in the multi-time domain analysis of the LYR, the real data were modified with Surfer 15 software, and the real-part isoline map of the bankfull discharge wavelet coefficients of the LYR from 1950 to 1999 was obtained (Fig. 4). The ordinate table is the time period, and the abscissa represents the study period (1950–1999). The image center point corresponding to the ordinate is the main period, and the difference between the two negative or positive years of the abscissa is the period. It can be seen in Fig. 4 that the real part of the wavelet coefficients shows obvious alternating positive and negative values over the entire time domain of about 26 years. The negative value area indicates that the bankfull discharge rate is relatively low, and the positive value area indicates that is relatively high of the LYR during this period. The zero value that appears in the graph, the junction between light and dark, is the turning point of the periodic change. As shown in Fig. 4b for the JHT station, the main period of  image center point 26 corresponding to the ordinate is 18 years, and the difference between the two negative values or two positive values of the abscissa corresponds to 18 years. In the picture, warm colors are positive, and cold colors are negative.

During the first phase (1950–1999) of the downstream of the Yellow River, there is a change rule of the first main period of about 26 years. The center value in Fig. 4 is consistent with the maximum value of Fig. 3a. In the meantime, these seven hydrological stations of the LYR also have a different second main period, but the second main period does not cover all time zones, and the light–dark alternation is not obvious, so it will not be repeated. Three oscillations with alternating positive and negative phases occur in the LYR over a time scale of about 26 years. The oscillation period is about 18 years, which is highly stable and is global over the entire time domain. At the same time, the oscillation characteristics in Fig. 4 are consistent with the trend map changes in the first main period of Fig. 3b.

### Evolution characteristics of bankfull discharge after XLDR operation began (2000–2020)

**Figure 5:**
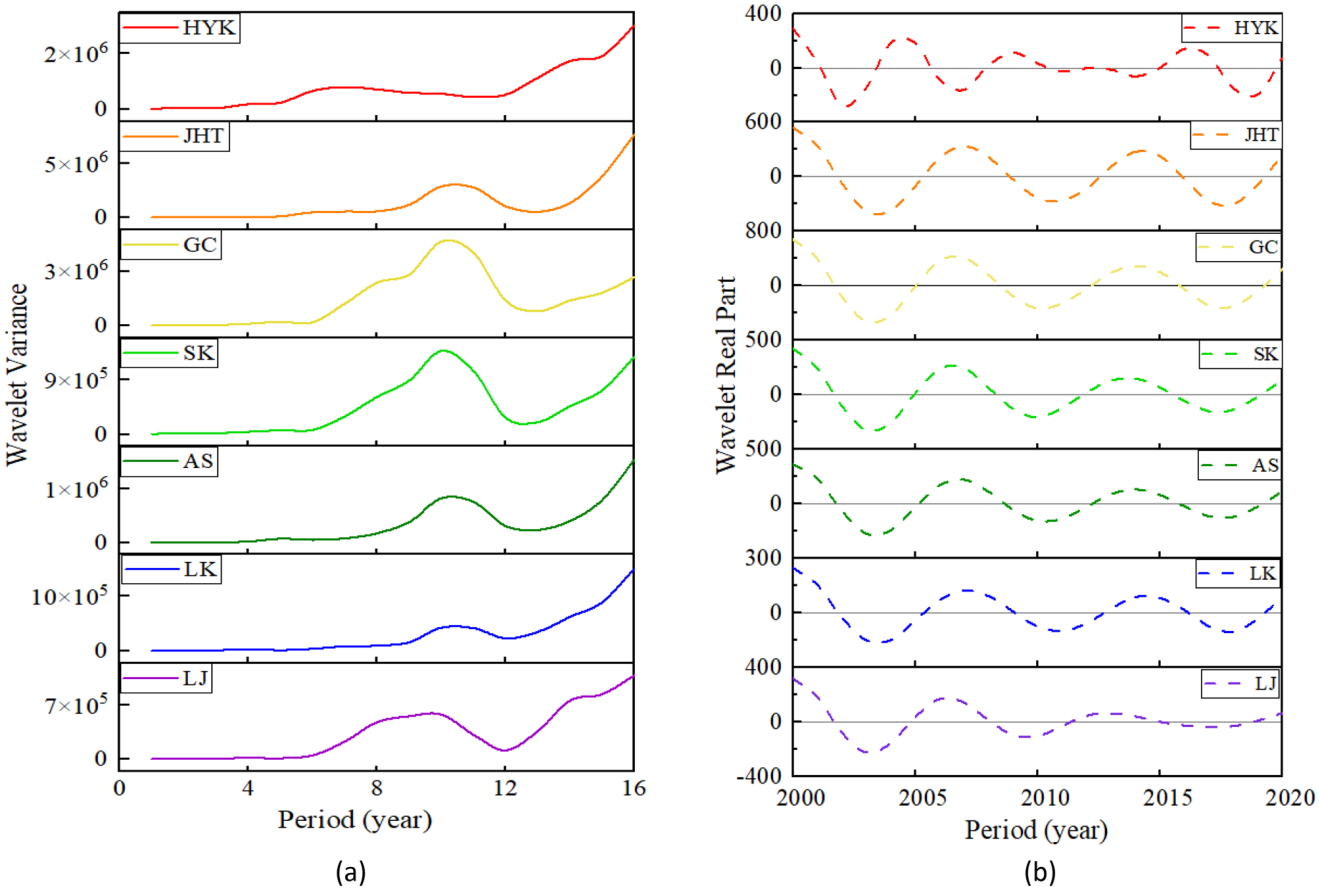
Variance diagram of wavelet coefficients for bankfull discharge of the LYR from 2000 to 2020: (**a**) wavelet variance, (**b**) main period trend.

Figure 5 is a variance diagram of the wavelet coefficients of the bankfull discharge of these seven hydrological stations of the LYR from 2000 to 2020. Figure 5a shows the variance of bankfull discharge, which reflects the main period of the evolution of bankfull discharge in this phase. Figure 5b is a trend chart of the main period of the bankfull discharge, which reflects the change of the real part of the wavelet coefficients under the first main period of the bankfull discharge, and defines the period under the first main period from 2000 to 2020 of the LYR.

In Fig. 5a, it can be seen that there is an obvious peak value, the maximum peak value, in the wavelet variance map of the bankfull discharge of the LYR from 2000 to 2020, corresponding to a time scale of about 10 years. These seven hydrological stations have an unnoticeable second peak value in different time scales, which is the second main period. It can be seen in Fig. 5b that, under the time scale of the first main period, the period of bankfull discharge of the LYR is about 7 years, and it experienced quasi-three oscillations. The evolution period of bankfull discharge in 2000–2020 of the LYR is shown in Table 3.

**Table 3:** Evolution period of bankfull discharge in the LYR from 2000 to 2020.

Period (Years)	Hydrological Station						
	HYK	JHT	GC	SK	AS	LK	LJ
First primary period	7	10	10	10	10	10	10
Period under the first main period	4–5	7–8	7–8	6–7	6–7	7–8	6–7

**Figure 6:**
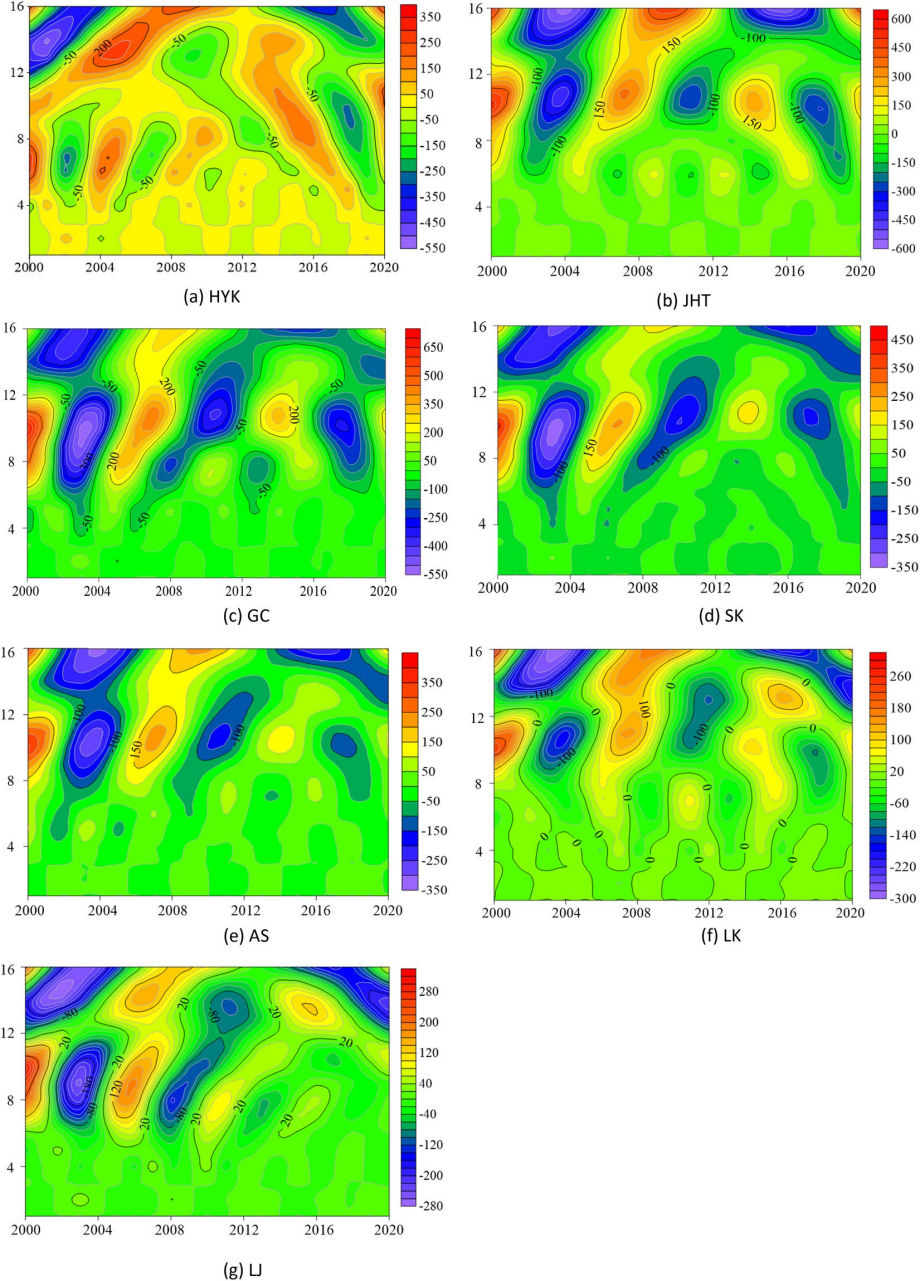
Real contour map of wavelet coefficients for the bankfull discharge of the LYR from 2000 to 2020. Created by MATLAB software (https://www.mathworks.com/products/matlab.html) and SUFER15 software (https://www.goldensoftware.com/products/surfer).

Figure 6 shows the contour map of the real part of the wavelet coefficient of the bank-full discharge in the LYR from 2000 to 2020. The ordinate table is the time period, and the abscissa represents the study period (2000–2020). The image center point corresponding to the ordinate is the main period, and the difference between the two negative or positive years of the abscissa is the period. It can be seen that the real part of the wavelet coefficients in the entire time domain shows an obvious positive and negative alternating change law over the time scale of about 10 years. The negative value area indicates that the bankfull discharge rate is relatively low, and the positive value area indicates that is relatively high of the LYR during this period. The zero value in the figure is the junction of light and dark, which is the turning point of periodic change. As shown in Fig. 6b for the JHT station, the main period of image center point 10 corresponding to the ordinate is 10 years, and the difference between the two negative values or two positive values of the abscissa corresponds to about 7 years. The warm color is positive, and the cool color is negative.

During the evolution of the bankfull discharge in the second phase (2000–2020) of the LYR, there is a variation law of the first main period of about 10 years. The central value in Fig. 6 is consistent with the maximum value of the wavelet variance diagram in Fig. 5a. At the same time, these stations in the LYR also show a different second main period at this phase. The second main period does not cover all areas, and the light and dark alternation is not obvious, so it also will not be repeated. The hydrologic stations in the LYR have experienced three oscillations with alternating positive and negative phases under a time scale of about 10 years. The oscillation period is about 7 years. It is highly stable over the entire time domain and has a global character. At the same time, the oscillation characteristics in Fig. 6 are consistent with the trend chart changes under the first main period in Fig. 5b.

The bankfull discharge of each hydrometric station in the LYR has a time scale of about 26 years in 1950–1999, and the period under this time scale is about 18 years. There is a time scale of about 10 years in the 2000–2020 long series years, and the period under this time scale is about 7 years. In order to explore the characteristics of different time scales and the periodic evolution trend of the bankfull discharge at the two phases in the LYR, the main factors affecting the evolution trend of the bankfull discharge were analyzed.

## Discussion

The LYR shows different discharge capacities under different water and sediment conditions, and the bankfull discharge of the river will change dynamically with the adjustment of discharge capacity. Therefore, the conditions of incoming water and sediment play a key role in the evolution trend of bankfull discharge. For that reason, this research focuses on the impact of the water and sediment conditions on the evolution of bankfull discharge.

### Variation process of annual runoff and sediment discharge

**Figure 7:**
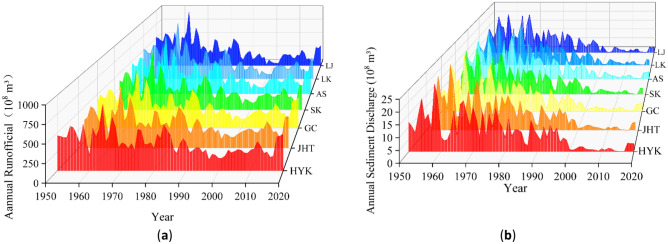
Variation of annual runoff and sediment discharge in the LYR from 1950 to 2020: (**a**) annual runoff, (**b**) annual sediment discharge. Created by ORIGIN software (https://www.originlab.com/).

Figure 7 shows the variation process of the annual runoff and sediment discharge of these seven hydrological stations of the LYR from 1950 to 2020. In Fig. 7, it can be seen that annual runoff and sediment discharge show obvious fluctuations, and their variation regularities are obviously different before and after 2000, which is consistent with the two phases of the evolution trend of bankfull discharge. Therefore, the variation process of Fig. 7 is also divided into two phases: before XLDR operation (1950–1999) and after XLDR operation began (2000–2020). Annual runoff and sediment discharge in the first phase vary greatly, and annual runoff and sediment discharge increase or decrease simultaneously. The difference between runoff and sediment discharge in the same year between 1950 and 1969 hydrological stations is small, and the difference in hydrological stations between 1970 and 1999 is obvious. The annual runoff of the LYR in the second phase increases first, then decreases slightly, and finally increases significantly. The annual sediment discharge in this phase begins to change more stably and then increases slightly, and the annual sediment discharge is greatly reduced compared with the first phase.

On the whole, in the first phase before XLDR operation, annual runoff and sediment discharge in 1960–1978 and 1978–1996 showed a cyclical change trend of dry–rich–dry. In the second phase, after the operation of the XLDR began, the annual runoff and sediment discharge in 2002–2009 and 2009–2016 also show a cyclical change trend of dry–rich–dry.

### Before XLDR operation (1950–1999)

Specific analysis shows that the LYR from 1950 to 1960 are a natural inflow and sediment period with large fluctuations and irregularities. The Sanmenxia Reservoir was put into operation in 1960. From September 1960 to October 1964, the clear water of the LYR discharged, resulting in scouring along the downstream river and the bankfull discharge increasing. The operation period of the Sanmenxia Reservoir from September 1960 to March 1962 consisted of water storage and sediment retention. From April 1962 to October 1964, the Sanmenxia Reservoir adopted a mode of flood detention and sediment retention. From November 1964 to October 1973, it continued to adopt this mode. During this period, the river course of the LYR was silted back, resulting in the oversaturated condition of the sediment coming from the LYR, and the sediment was continuously silted along the course. At the same time, serious interruptions occurred in the LYR. The annual runoff continued to decrease, and the bankfull discharge also decreased. From 1974 to 1985, the Sanmenxia Reservoir adopted an operation mode of clean storage and turbidity removal, discharging clean water during non-flood seasons and scouring downstream rivers. During flood seasons, reservoir sediment discharge, and sediment reduction, the flow capacity of the main channel increased, and the bankfull discharge showed an increasing trend as a whole. The annual runoff and sediment discharge reached maximum values near 1978. The uneven distribution of water and sediment of the LYR from 1986 to 1999 aggravated the rise of the sediment deposition of the LYR. From 1990 to 1996, the upper reaches of the Yellow River experienced seven consecutive years of dry season, which resulted in the serious shrinkage of the lower reaches. The Sanmenxia Reservoir continued the operation mode of clean storage and turbidity removal. The Longyangxia Reservoir has been regulated for many years, but it was still unable to avoid the continued interruption of the LYR. The downstream channel shrank severely, and the flow capacity of the river decreased, the bankfull discharge decreased continuously during this period. The annual runoff and sediment discharge reached minimum values near 1999.

### After the operation of the XLDR began (2000–2020)

The XLDR was put into operation in 2000. At the beginning of the XLDR impounding and sediment retention from 2000 to 2001, the scouring efficiency of the LYR was not high, and the flow rate of the bankfull discharge only increased slightly. From 2002 to 2007, the XLDR was in an efficient operation period for water and sediment regulation. As the sediment concentration of reservoir sediment detention in the downstream decreased sharply, and the annual runoff of these seven hydrological stations was increasing, the river channel was greatly scoured, the flow capacity of the main channel was increased, and the bankfull discharge increased accordingly. After 2008, some reaches of the LYR became silted. Although the scouring efficiency of the XLDR during water and sediment diversion was slightly reduced, the scouring of most reaches could be guaranteed, and the bankfull discharge still increased steadily. The annual runoff and sediment discharge reached maximum values near 2009. From 2000 to 2016, sediment discharge capacity of the XLDR was low, sediment deposition in the reservoir increased, and annual runoff and sediment discharge both decreased, resulting in the reduced flow capacity of the downstream rivers and the reduced bankfull discharge. The annual runoff and sediment discharge reached maximum values near 2016. After 2018, the LYR was in flood season, annual runoff increased substantially, annual sediment discharge increased slightly, and annual runoff and annual sediment discharge reached maximum values in the second phase, with an increase in bankfull discharge.

### Response of bankfull discharge to water and sediment conditions

In this research, the scouring intensity $F_{{\text{i }}}$ is used to reflect the response of the bankfull discharge to the inflow conditions^21^. $F_{i} = (\overline{{Q_{i} }}^{2} /\overline{{S_{i} }} )/10^{4}$, where $F_{{\text{i }}}$ is the average flow scouring intensity, m^9^/kg/s^2^, $\overline{Q}_{{\text{i }}}$ is the average flow in year i, m^3^/s, and $\overline{S}_{{\text{i }}}$ is the average sediment concentration in year i, kg/m^3^.

**Figure 8:**
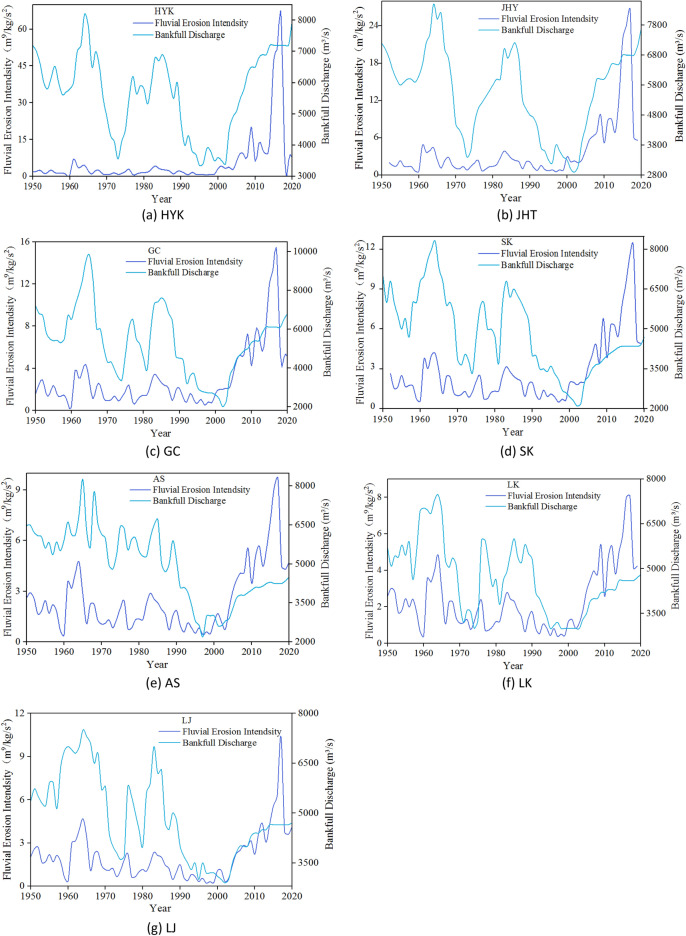
Variation process of scouring intensity in the LYR.

Figure 8 shows the change process of the bankfull discharge and flow scouring intensity of the seven hydrological stations in the LYR from 1950 to 2020. Figure 8 shows that the bankfull discharge of the LYR increased with the increase in flow scouring intensity and decreased with the decrease in flow scouring intensity, which is a positive correlation. From the point of view of distance and correlation degree, these seven hydrological stations of the LYR have increasingly strong correlations with the increase in distance along the river.

In this research, the sediment inflow coefficient $\xi$ is used to reflect the response of flat discharge to sediment inflow conditions^33^. $\xi = \overline{S} /\overline{Q}$, where $\xi$ is the sediment inflow coefficient, kg s/m^6^, $\overline{S}$ is the average sediment concentration, kg/m^3^, and $\overline{Q}$ is the average flow, m^3^/s.

**Figure 9:**
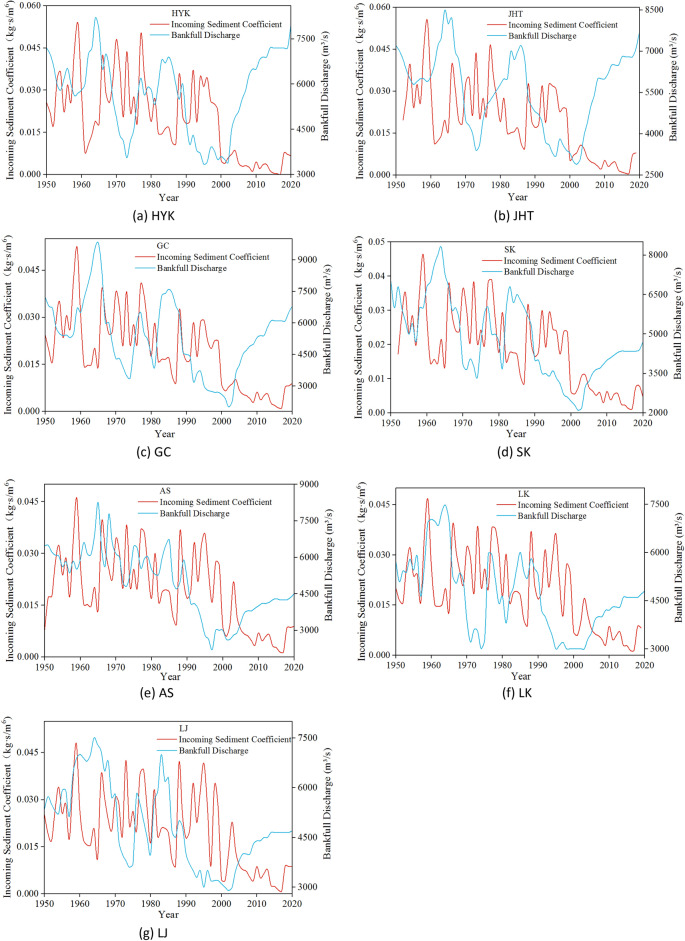
Variation process of sediment coefficient in the LYR.

Figure 9 shows the variation process of the bankfull discharge and sediment coefficient of these seven hydrological stations in the LYR from 1950 to 2020. In Fig. 9, it can be seen that the bankfull discharge of the LYR decreased with the increase in sediment coefficient and decreased with the increase in sediment coefficient, which is a negative correlation. From the point of view of the course, the correlation of the LYR becomes increasingly strong with the increase in the distance along the course. The fluctuation amplitude of the bankfull discharge is smaller than that of the coefficient of sediment inflow, which reflects the fact that the bankfull discharge lags behind the variation in sediment inflow conditions.

The results show that the scouring and silting changes of the LYR are closely related to the scouring intensity of currents and the coefficient of sediment inflow. When the scouring intensity of currents is high or the coefficient of sediment inflow is low, the bankfull discharge will increase accordingly. When the intensity of water flow scouring is small or the coefficient of incoming sediment is high, the channel silts and the bankfull discharge decreases accordingly.

## Conclusion

1. According to the evolution trend of bankfull discharge in the LYR from 1950 to 2020, the research period was divided into two phases according to the evolution characteristics of bankfull discharge: before the first phase of XLDR operation (1950–1999), the bankfull discharge shows an alternating increasing and decreasing trend. After operation of the XLDR began in the second phase (2000–2020), the bankfull discharge continued to increase.
2. Based on wavelet analysis, before the XLDR (1950–1999), the time scale reflecting the bankfull discharge of the LYR is about 26 years, and its evolution period is 18 years under this time scale. After XLDR operation began (2000–2020), the time scale reflecting the bankfull discharge of the LYR is about 10 years, and its evolution period is about 7 years under this time scale. This indicates that the bankfull discharge of the LYR has time-scale evolution characteristics.
3. The bankfull discharge of the LYR is mainly affected by incoming water and sediment conditions. Bankfull discharge increases with the increase in flow scouring intensity and decreases with the increase in the flow scouring coefficient, it is positively correlated with flow scouring intensity and negatively correlated with the flow scouring coefficient.

## Data Availability

The data presented in this study are available on request from the corresponding author.
